# Endoscopic Submucosal Dissection vs. Surgery for Superficial Esophageal Squamous Cancer: A Systematic Review and Meta-Analysis

**DOI:** 10.3389/fonc.2022.816832

**Published:** 2022-04-21

**Authors:** Zhifeng Liu, Renping Zhao

**Affiliations:** ^1^ Department of Gastroenterology, the Third People’s Hospital of Hubei Province, Jianghan University, Wuhan, China; ^2^ Department of Radiology, Wuhan Children’s Hospital (Wuhan Maternal and Child Healthcare Hospital), Tongji Medical College, Huazhong University of Science & Technology, Wuhan, China

**Keywords:** esophageal cancer, endoscopic submucosal dissection, surgery, morbidity, mortality, overall survival

## Abstract

**Background:**

Esophageal cancer is one of the leading causes of morbidity and mortality across the world. Only one systematic review and meta-analysis has attempted to compare the morbidity and mortality outcomes in superficial esophageal squamous cancer patients undergoing endoscopic submucosal dissection (ESD) and esophagectomy (ESO), but with several limitations. This study aimed at comparing the outcomes of hospital stay duration, procedure duration, recurrence, complications, all-cause mortality, short-term survival, and long-term survival in patients with superficial esophageal squamous cancer undergoing ESD and ESO.

**Methods:**

Six databases (Web of Science, PubMed, EMBASE, CENTRAL, Scopus, and MEDLINE) were systematically searched according to PRISMA guidelines for eligible studies. With the available literature, we conducted a random-effect meta-analysis to evaluate weighted effect size and odds ratios to determine the comparative morbidity and mortality outcomes between patients with superficial esophageal squamous cancer undergoing ESD and ESO.

**Results:**

We found 16 eligible studies detailing 5,213 and 8,049 age- and sex-matched patients undergoing ESD and ESO, respectively. Meta-analysis revealed reduced hospital stay (Hedge’s g: -1.22) and procedure duration (g: -4.54) for patients undergoing ESD. We also observed significantly reduced risks for complications (odds ratio: 0.35) and all-cause mortality (OR: 0.56) in patients undergoing ESD. Differences in recurrence (OR: 0.95), short-term outcomes (OR: 1.10), and long-term survival (OR: 0.81) outcomes were not significantly different between ESD and ESO.

**Conclusions:**

This meta-analysis provides evidence concerning the improved morbidity and mortality outcomes in superficial esophageal squamous cancer patients undergoing ESD as compared to ESO. The findings herein may aid in developing clinical awareness and assisting best practice guideline development for managing superficial esophageal squamous cancer.

**Registration:**

PROSPERO, https://www.crd.york.ac.uk/prospero/#searchadvanced, CRD42021286212.

## Introduction

Esophageal cancer is the seventh most common type of cancer across the world (1). Recent epidemiological data have reported poor long-term survival rates globally for patients with esophageal cancer (2), and the Global Burden of Disease study acknowledges that esophageal cancer is a strong predictor of mortality (3).

Conventionally, the management of superficial squamous esophageal cancer is carried out with esophagectomy (ESO) (4). However, studies over the past decades have widely associated the use of this radical procedure with high rates of intra-operative, postoperative complications and mortality (5–7). To circumvent this morbidity and mortality-related burden, recent studies have recommended the use of advanced minimally invasive endoscopic procedures such as endoscopic submucosal dissection (ESD) (8, 9). The procedure allows the removal of cancerous tissue with the help of a flexible endoscopic tube and can even be conducted during outpatient visits (10). Hanaoka, Tanabe et al. (11) mentioned that in addition to offering the therapeutic benefits, ESD also pertained to a diagnostic edge as its use was less limited because of the size of the lesion and that it permitted en-bloc with complete histological removal of lesions (12).

To date, several retrospective cohort studies have attempted to compare the morbidity- and mortality-related outcomes between patients with superficial esophageal squamous cancer [i.e., carcinoma limited to mucosa or submucosa regardless of lymph node status (13)] undergoing ESD and ESO (14–20). However, a consensus in the literature on this topic is currently absent. For instance, some studies in the existing literature have reported higher risks of recurrence in patients undergoing ESD relative to ESO (15, 16, 21), whereas other studies had contrarily reported higher recurrence risks for ESO as compared to ESD (14, 22, 23). Likewise, a consensus also lacks regarding the outcomes of long-term survival.

To the best of our knowledge, only one systematic review and meta-analysis to date has attempted to compare the morbidity- and mortality-related risks of ESO and ESD in patients with superficial esophageal squamous cancer (24). However, this study was limited in two regards. First, the authors did not explore the differences between the two procedures with respect to the duration of the procedure and the duration of hospital stay. Second, several relevant high-quality retrospective cohort studies were not included in the original meta-analysis (14, 16, 18, 19, 25).

We, therefore, in this systematic review and meta-analysis, attempt to bridge the knowledge gap pertaining to the comparative differences in terms of hospital stay duration, procedure duration, recurrence, complications, all-cause mortality, short-term survival, and long-term survival in patients with superficial esophageal squamous cancer undergoing ESD and ESO. Our findings will hopefully elevate clinical awareness and understanding of the morbidity- and mortality-related risks associated with the management of superficial esophageal squamous cancer.

## Methods

We adhered to the PRISMA (Preferred Reporting Items for Systematic Reviews and Meta-Analyses) guidelines (26) for performing this meta-analysis. This study was preregistered at PROSPERO (No. CRD42021286212).

### Data Search Strategy

The literature search was performed in six scientific databases (Web of Science, PubMed, MEDLINE, CENTRAL, EMBASE, and Scopus) from inception till September 2021. The search was performed across a combination of MeSH keywords including “squamous cell carcinoma,” “superficial esophageal cancer,” “esophagectomy,” “endoscopic submucosal dissection,” “morbidity,” “hospital stay,” “procedure time,” “metastasis,” “complications,” “recurrence,” “overall survival,” and “mortality.” The reference sections for included studies were manually scanned to identify additional relevant studies.

The inclusion criteria were as follows:

- Studies comparing the outcomes of hospital stay duration, procedure duration, recurrence, complications, all-cause mortality, short-term survival, and long-term survival in patients with superficial esophageal squamous cancer undergoing ESD and ESO;
- Studies with human participants;
- Case–control studies, prospective trials, or retrospective cohort trials;
- Studies published in peer-reviewed scientific journals;
- Studies published in English.

Screening was performed by two reviewers independent of each other. Disagreements were resolved through discussion between the two reviewers.

### Quality Assessment

Bias risk for each included study was assessed using the Newcastle–Ottawa scale (27). This tool evaluates the study outcomes for selective reporting, confounding bias, measurement of outcomes, and incomplete data availability. Methodological quality was appraised, again, by two reviewers working independently of each other. Again, the two reviewers arbitrated in case of a dispute.

### Primary Outcomes

The primary outcomes evaluated in the study determined the overall incidence of all-cause mortality, short-term survival, and long-term survival in patients with esophageal squamous cancer.

### Secondary Outcomes

The primary outcomes evaluated in the study determined the overall incidence of recurrence procedure duration, hospital stay duration, and complications in patients with esophageal squamous cancer.

### Data Analysis

We performed a within-group meta-analysis using Comprehensive Meta-analysis (CMA) software version 2.033 based on a random-effect model. We calculated the weighted effect size to determine the hospital stay duration and procedure time. We also calculated odds ratios to determine the overall incidence of recurrence, complications, all-cause mortality, short-term survival, and long-term survival in patients with esophageal squamous cancer. Furthermore, we assessed study heterogeneity using I^2^ statistics (0%–25%: negligible heterogeneity, 25%–75%: moderate heterogeneity, ≥75%: substantial heterogeneity). Publication bias was evaluated using Duval and Tweedie’s trim-and-fill procedure. The significance level for this study was set at 5%.

## Results

**Figure 1:**
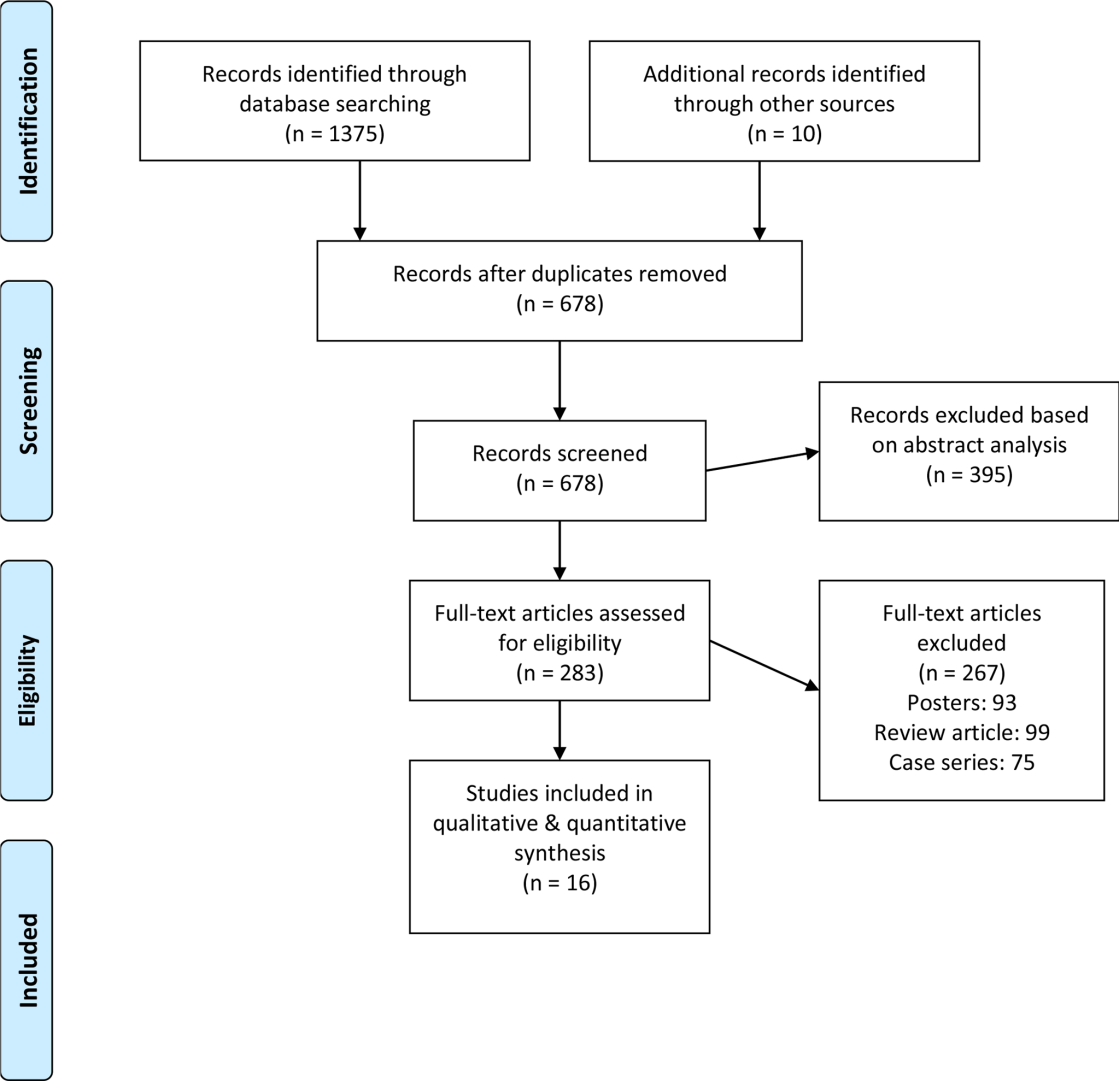
Illustrating the PRISMA flowchart.

Initial database scanning yielded 1,375 studies. A further 10 studies have been added to this total when screening reference sections from included studies. From this, 16 studies remained after applying inclusion criteria (**Figure 1**). All of these were retrospective cohort studies (**Table 1**).

**Table 1:** Details of the included studies.

Study	Country	Type	Sample descriptive	Age (M ± S.D years)	Hospital stays	Procedure time (min)	Short-term survival rate	Long-term survival rate	All-cause mortality (n)	Recurrence/metastasis (n)	Complications (n)
Wang, Chen et al. (19)	China	RCS	ESD: 217 (41F, 176M) ESO: 217 (34F, 183M)	–	–	–	–	5-year ESD: 66.1 ESO: 79.3	–	–	–
Yamauchi, Iwamuro et al. (20)	Japan	RCS	ESD: 50 ESO: 50	ESD: 69 ESO: 65	–	–	–	5-year ESD: 84.5 ESO: 79	ESD: 8 ESO: 9	ESD: 3 ESO: 3	ESD: 15 ESO: 19
Kamarajah, Markar et al. (18)	United Kingdom	RCS	ESD: 1581 (260F, 1321M) ESO: 964 (144F, 820M)	60	–	–	3-year ESD: 83 ESO: 85	5-year ESD: 70 ESO: 74	ESD: 16 ESO: 40	–	–
McCarty, Parker et al. (17)	USA	RCS	ESD: 772 (146F, 626M) ESO: 1361 (199F, 1162)	ESD: 68.4 ± 10.5 ESO: 63.6 ± 9.5	–	–	–	5-year ESD: 78.5 ESO: 77.4	ESD: 199 ESO: 413	–	–
An, Liu et al. (16)	China	RCS	ESD: 222 (65F, 157M) ESO: 184 (59F, 125M)	ESD: 67.8 ± 8.3 ESO: 66.8 ± 7.1	ESD: 3 ESO: 10	ESD: 80 ESO: 260	–	5-year ESD: 81 ESO: 81	ESD: 24 ESO: 26	ESD: 28 ESO: 26	ESD: 74 ESO: 70
Zhang, Ding et al. (15)	China	RCS	ESD: 322 (64F, 258M) ESO: 274 (81F, 193M)	ESD: 63.5 ± 8.3 ESO: 62.3 ± 7.4	ESD: 3 ESO: 11	ESD: 49 ESO: 240	3-year ESD: 91 ESO: 87.7	4-year ESD: 82.9 ESO: 79.3	ESD: 22 ESO: 28	ESD: 27 ESO: 23	ESD: 49 ESO: 76
Yuan, Liu et al. (14)	China	RCS	ESD: 69 (18F, 51M) ESO: 47 (11F, 36M)	ESD: 63.7 ESO: 61.1	ESD: 10.7 ESO: 19.2	–	3-year ESD: 98.6 ESO: 93.6	5-year ESD: 97.1 ESO: 91.5	ESD: 0 ESO: 2	ESD: 6 ESO: 0	ESD: 30 ESO: 34
Gong, Yue et al. (25)	China	RCS	ESD: 78 (13F, 65M) ESO: 128 (23F, 105M)	ESD: 60.5 ± 8.7 ESO: 59.5 ± 7	ESD: 10.1 ± 7.9 ESO: 24.1 ± 9.4	–	–	–	–	–	ESD: 18 ESO: 31
Qin, Peng et al. (28)	China	RCS	ESD: 224 (50F, 174M) ESO: 196 (40F, 156M)	–	–	–	–	5-year ESD: 39.6 ESO: 50.5	ESD: 36 ESO: 42	–	–
Takeuchi, Suda et al. (21)	Japan	RCS	ESD: 65 ESO: 54	ESD: 68 ESO: 64	ESD: 31 ESO: 34.5	ESD: 502 ESO: 552	–	–	–	ESD: 1 ESO: 5	ESD: 5 ESO: 17
Min, Lee et al. (29)	Korea	RCS	ESD: 120 (9F, 111M) ESO: 120 (9F, 111M)	ESD: 63.9 ± 8.0 ESO: 63.3 ± 8.1	–	–	3-year ESD: 95.7 ESO: 92.6	5-year ESD: 94 ESO: 87.1	ESD: 8 ESO: 27	–	ESD: 29 ESO: 106
Zeng, Liang et al. (30)	China	RCS	ESD: 738 (149F, 589M) ESO: 1923 (283F, 1640M)	–	–	–	3-year ESD: 77.5 ESO: 85.1	5-year ESD: 65.1 ESO:79.7	–	–	–
Li, Yamashita et al. (23)	Canada	RCS	ESD: 11 (4F, 7M) ESO: 12 (2F, 10M)	ESD: 65.3 ± 12 ESO: 64.8 ± 8.8	ESD: 0 ESO: 10	–	–	–	–	ESD: 1 ESO: 1	ESD: 11 ESO: 12
Jin, Gai et al. (22)	China	RCS	ESD: 59 (23F, 36M) ESO: 40 (12F, 28M)	–	ESD: 6 ± 3.8 ESO: 19 ± 8	ESD: 74 ± 23 ESO: 294 ± 46	3-year ESD: 96.6 ESO: 97.5	4-year ESD: 91.5 ESO: 90	ESD: 0 ESO: 0	ESD: 5 ESO: 2	ESD: 8 ESO: 12
Cummings, Kou et al. (31)	USA	RCS	ESD: 255 (58F, 197M) ESO 893 (202F, 691M)	–	–	–	2-year ESD: 84 ESO: 71	–	–	ESD: 32 ESO: 139	ESD: 30 ESO: 265
Wani, Drahos et al. (32)	USA	RCS	ESD: 430 (95F, 335M) ESO: 1586 (123F, 1463M)	ESD: 70.5 ± 10.3 ESO: 63.4 ± 9.8	–	–	2-year ESD: 78.4 ESO: 81.8	5-year ESD: 16.7 ESO: 36.6	ESD: 55 ESO: 310	–	–

M, mean; S.D, standard deviation; F, female; M, male; RCS, retrospective cohort study; ESD, endoscopic submucosal dissection; ESO, esophagectomy.

### Participant Information

The 16 included studies contained data on 13,262 patients. Among these, 5,213 underwent ESD, whereas 8,049 patients underwent ESO. The average age of participants undergoing ESD was 65.5 ± 3.4 years, and the average age of patients undergoing ESO was 63 ± 2.2 years. Five studies did not report patient age information (19, 22, 28, 30, 31).

### Quality Assessment Cohort Studies

**Figure 2:**
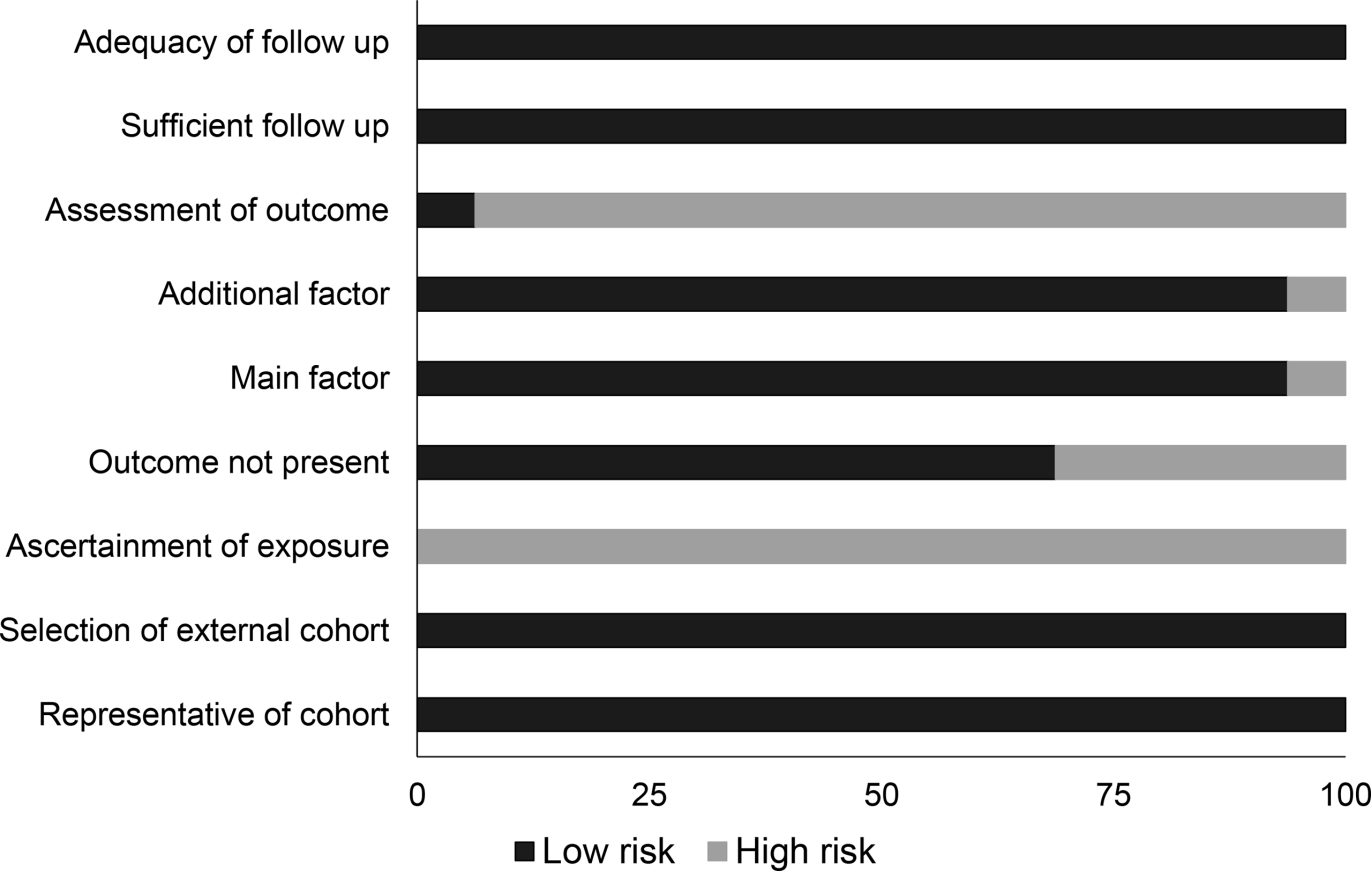
Demonstrates the risk of bias according to the Newcastle–Ottawa scale for cohort studies.

The Newcastle–Ottawa scale showed that overall bias risk was low in all the included studies (**Table 2** and **Figure 2**). We observed that lack of adequate assessment of outcome and ascertainment of exposure were the most predominant aspects of concern.

**Table 2:** Risk of bias for individual studies based on the Newcastle–Ottawa scale (+: low risk of bias, 0: high risk of bias).

Study	Selection				Comparability		Outcome			Total
	Representative of the exposed cohort	Selection of external cohort	Ascertainment of exposure	Outcome of interest does not present at start	Main factor	Additional factor	Assessment of outcome	Sufficient follow-up	Adequacy of follow-up	(9/9)
Wang, Chen et al. (19)	+	+	0	+	+	+	0	+	+	7
Yamauchi, Iwamuro et al. (20)	+	+	0	0	+	+	0	+	+	6
Kamarajah, Markar et al. (18)	+	+	0	+	+	0	+	+	+	7
McCarty, Parker et al. (17)	+	+	0	+	+	+	0	+	+	7
An, Liu et al. (16)	+	+	0	+	+	+	0	+	+	7
Zhang, Ding et al. (15)	+	+	0	+	+	+	0	+	+	7
Yuan, Liu et al. (14)	+	+	0	0	+	+	0	+	+	6
Gong, Yue et al. (25)	+	+	0	0	0	+	0	+	+	5
Qin, Peng et al. (28)	+	+	0	+	+	+	0	+	+	7
Takeuchi, Suda et al. (21)	+	+	0	+	+	+	0	+	+	7
Min, Lee et al. (29)	+	+	0	+	+	+	0	+	+	7
Zeng, Liang et al. (30)	+	+	0	0	+	+	0	+	+	6
Li, Yamashita et al. (23)	+	+	0	+	+	+	0	+	+	7
Jin, Gai et al. (22)	+	+	0	+	+	+	0	+	+	7
Cummings, Kou et al. (31)	+	+	0	+	+	+	0	+	+	7
Wani, Drahos et al. (32)	+	+	0	0	+	+	0	+	+	6

### Publication Bias

**Figure 3:**
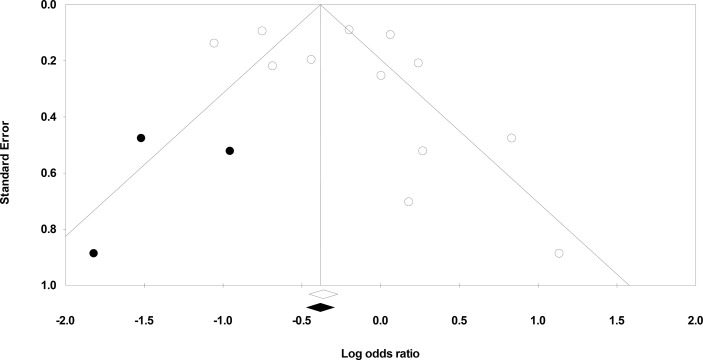
Demonstrates the publication bias by Duval & Tweedy’s trim and fill method.

We used Duval and Tweedie’s trim-and-fill method to identify whether studies are missing from this meta-analysis on either side of the mean effect (33). This method found that three studies were missing on the left side of the mean effect. The overall random-effect model determined the point estimate and 95% confidence interval for all studies combined to be 0.81 (0.60 to 1.09). However, with the trim and fill the imputed estimate is 0.71 (0.53 to 0.94) (**Figure 3**).

### Meta-Analysis Report

#### Hospital Stay Duration

**Figure 4:**
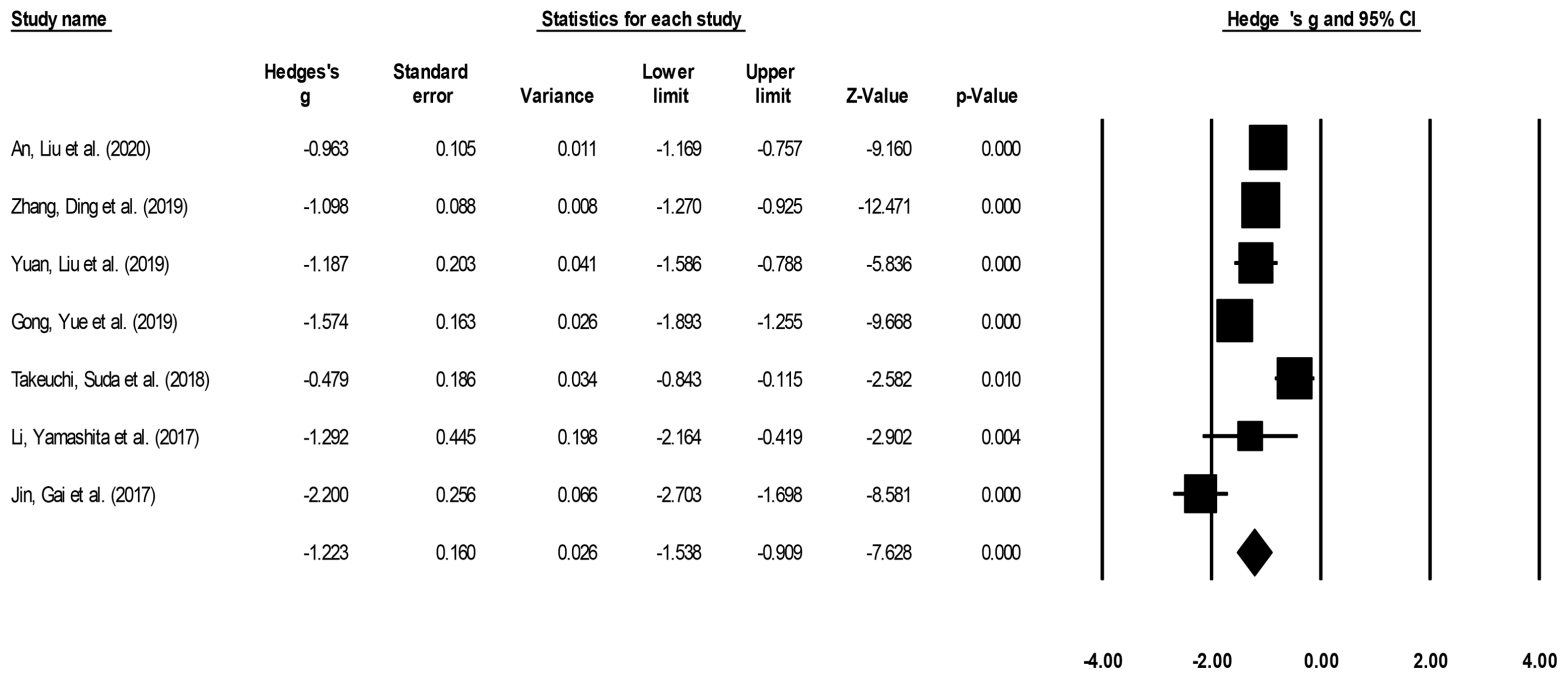
Demonstrates the forest plot for studies evaluating the hospital stay duration in patients with superficial esophageal squamous cancer undergoing endoscopic submucosal dissection or esophagectomy. The weighted effect sizes are presented as black boxes whereas 95% confidence intervals are presented as whiskers. A positive effect size represents a shorter hospital duration for patients receiving esophagectomy, and a negative effect size represents a shorter hospital duration for patients receiving endoscopic submucosal dissection.

Hospital stay duration was reported by seven studies (14–16, 21–23, 25). We noted a large effect, negative effect size for hospital stay duration in patients undergoing ESD relative to patients undergoing ESO (**Figure 4**) (Hedge’s g: -1.22, 95% CI: -1.53 to -0.90, p < 0.001) with moderate heterogeneity (I^2^: 34.9%).

#### Procedure Duration

**Figure 5:**
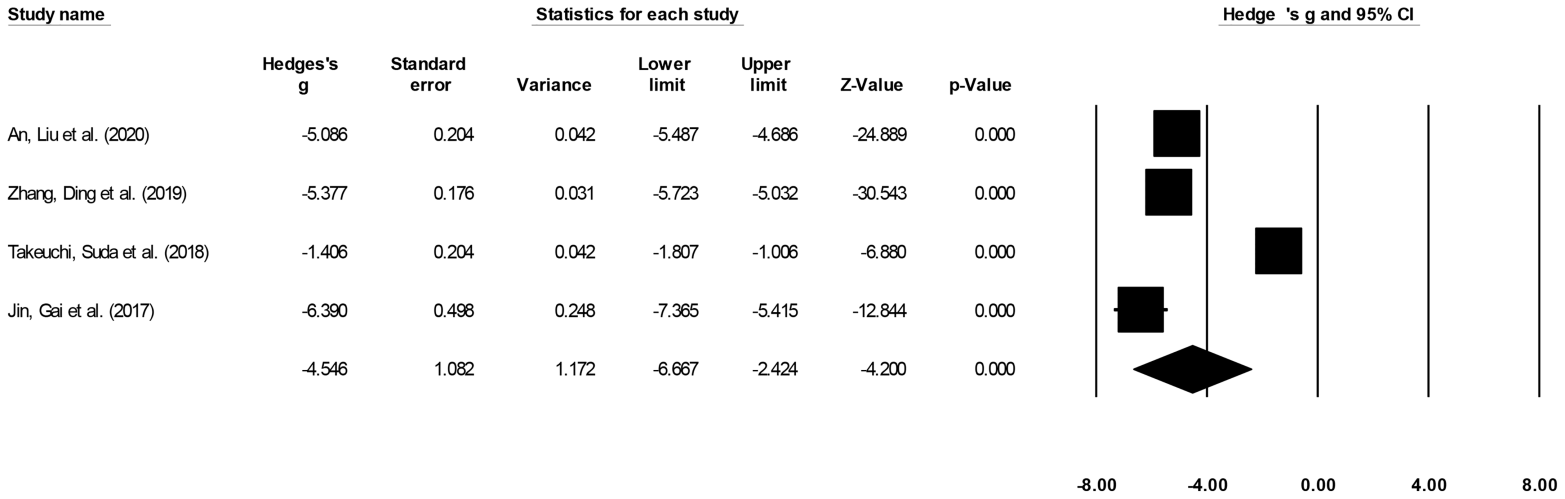
The forest plot for studies evaluating the procedure duration in patients with superficial esophageal squamous cancer undergoing endoscopic submucosal dissection or esophagectomy. The weighted effect sizes are presented as black boxes whereas 95% confidence intervals are presented as whiskers. A positive effect size represents a shorter procedure duration for patients receiving esophagectomy, and a negative effect size represents a shorter procedure duration for patients receiving endoscopic submucosal dissection.

Procedure duration was reported by four studies (15, 16, 21, 22). We noted a large effect, negative effect size for procedure duration in patients undergoing ESD relative to patients undergoing ESO (**Figure 5**) (Hedge’s g: -4.54, 95% CI: -6.66 to -2.42, p < 0.001) with negligible heterogeneity (I^2^: 1.2%).

#### Recurrence

**Figure 6:**
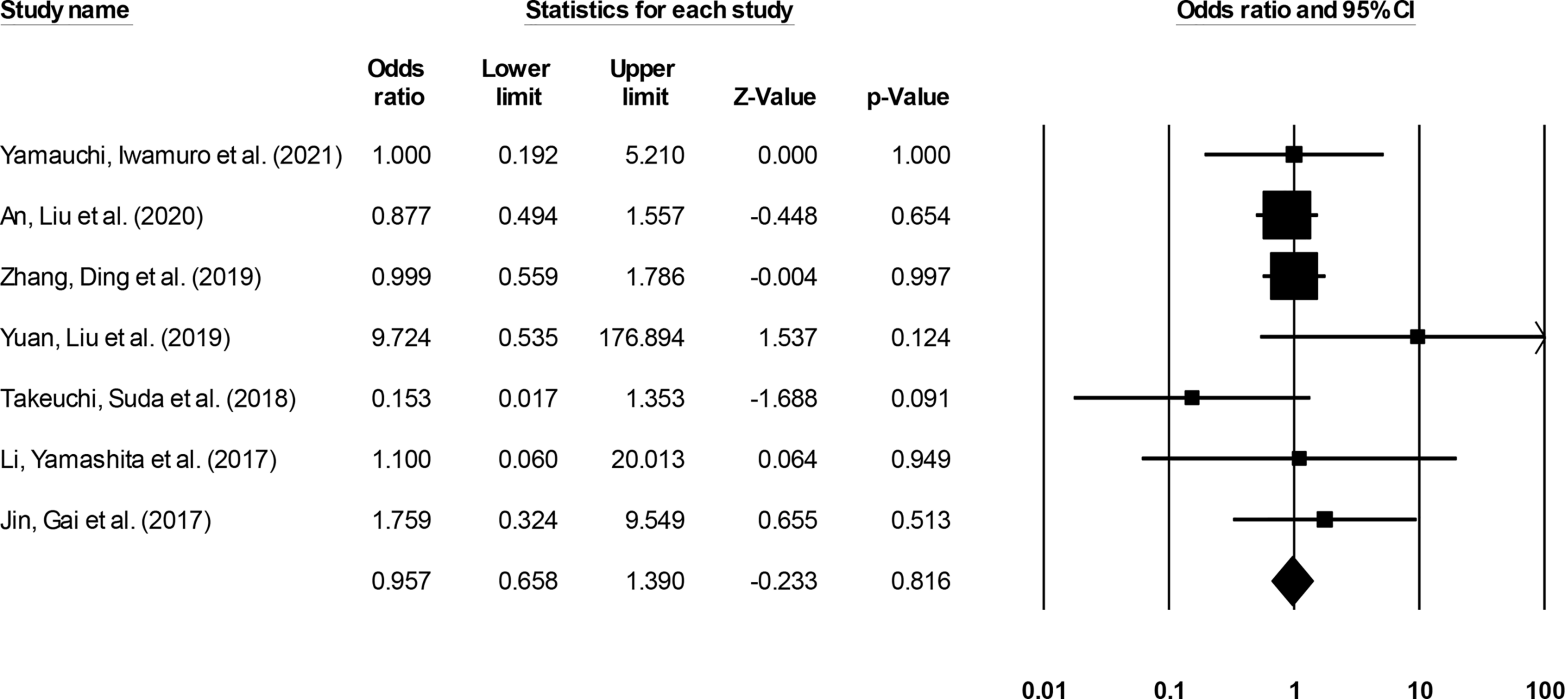
The forest plot for studies evaluating the recurrence incidence in patients with superficial esophageal squamous cancer undergoing endoscopic submucosal dissection or esophagectomy. The odds ratios are presented as black boxes whereas 95% confidence intervals are presented as whiskers. A lower odds ratio represents higher risks of recurrence for patients receiving endoscopic submucosal dissection, and a higher odds ratio represents higher risks of recurrence for patients receiving esophagectomy.

Recurrence incidence was reported by seven studies (14–16, 20–23). We noted no significant differences in the odds for recurrence events in patients undergoing ESD relative to patients undergoing ESO (**Figure 6**) (0.95, 95% CI: 0.65 to 1.39, p = 0.81) with no heterogeneity (I^2^: 0%).

#### Complications

**Figure 7:**
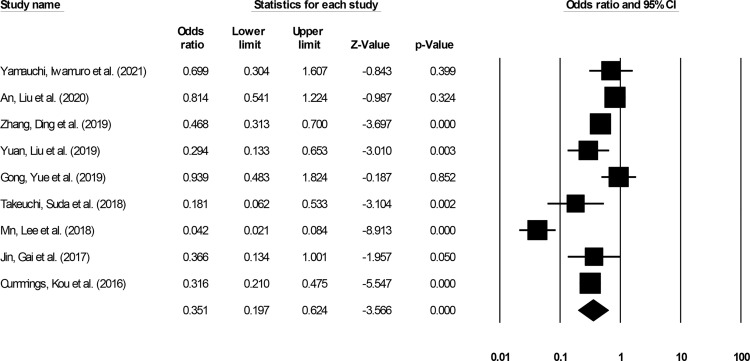
The forest plot for studies evaluating the complication incidence in patients with superficial esophageal squamous cancer undergoing endoscopic submucosal dissection or esophagectomy. The odds ratios are presented as black boxes whereas 95% confidence intervals are presented as whiskers. A lower odds ratio represents higher risks of complications for patients receiving endoscopic submucosal dissection, and a higher odds ratio represents higher risks of complications for patients receiving esophagectomy.

Complication incidence was reported by nine studies (14–16, 20–22, 25, 29, 31). We noted significantly reduced odds for complication events in patients undergoing ESD relative to patients undergoing ESO (**Figure 7**) (0.35, 95% CI: 0.19 to 0.62, p < 0.001) with minimal heterogeneity (I^2^: 14.5%).

#### All-Cause Mortality

**Figure 8:**
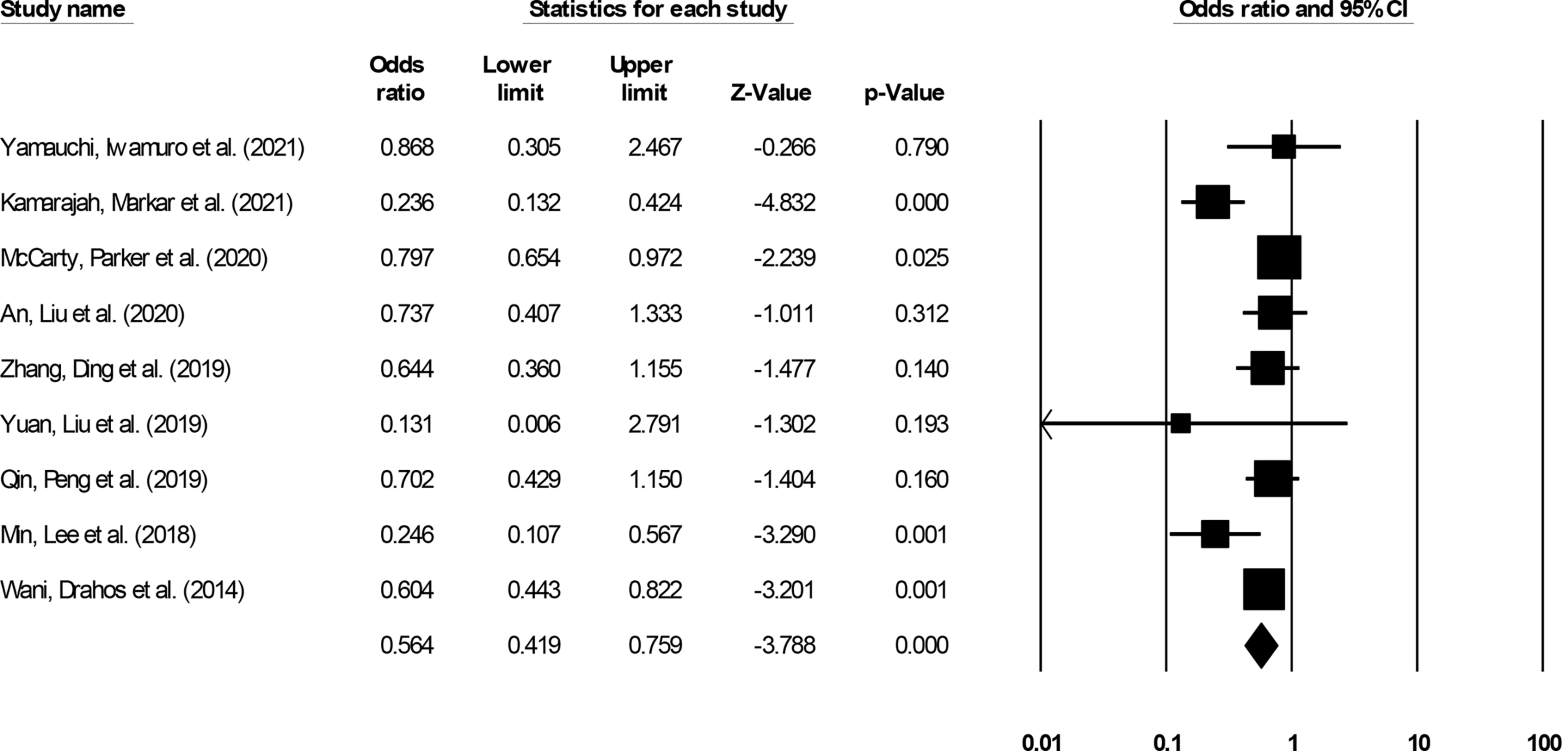
The forest plot for studies evaluating the all-cause mortality incidence in patients with superficial esophageal squamous cancer undergoing endoscopic submucosal dissection or esophagectomy. The odds ratios are presented as black boxes whereas 95% confidence intervals are presented as whiskers. A lower odds ratio represents higher risks of all-cause mortality for patients receiving endoscopic submucosal dissection, and a higher odds ratio represents higher risks of all-cause mortality for patients receiving esophagectomy.

All-cause mortality incidence was reported by nine studies (14–18, 20, 28, 29, 32). We noted significantly reduced odds for all-cause mortality events in patients undergoing ESD relative to patients undergoing ESO (**Figure 8**) (0.56, 95% CI: 0.41 to 0.75, p < 0.001) with minimal heterogeneity (I^2^: 14.5%).

#### Short-Term Survival

**Figure 9:**
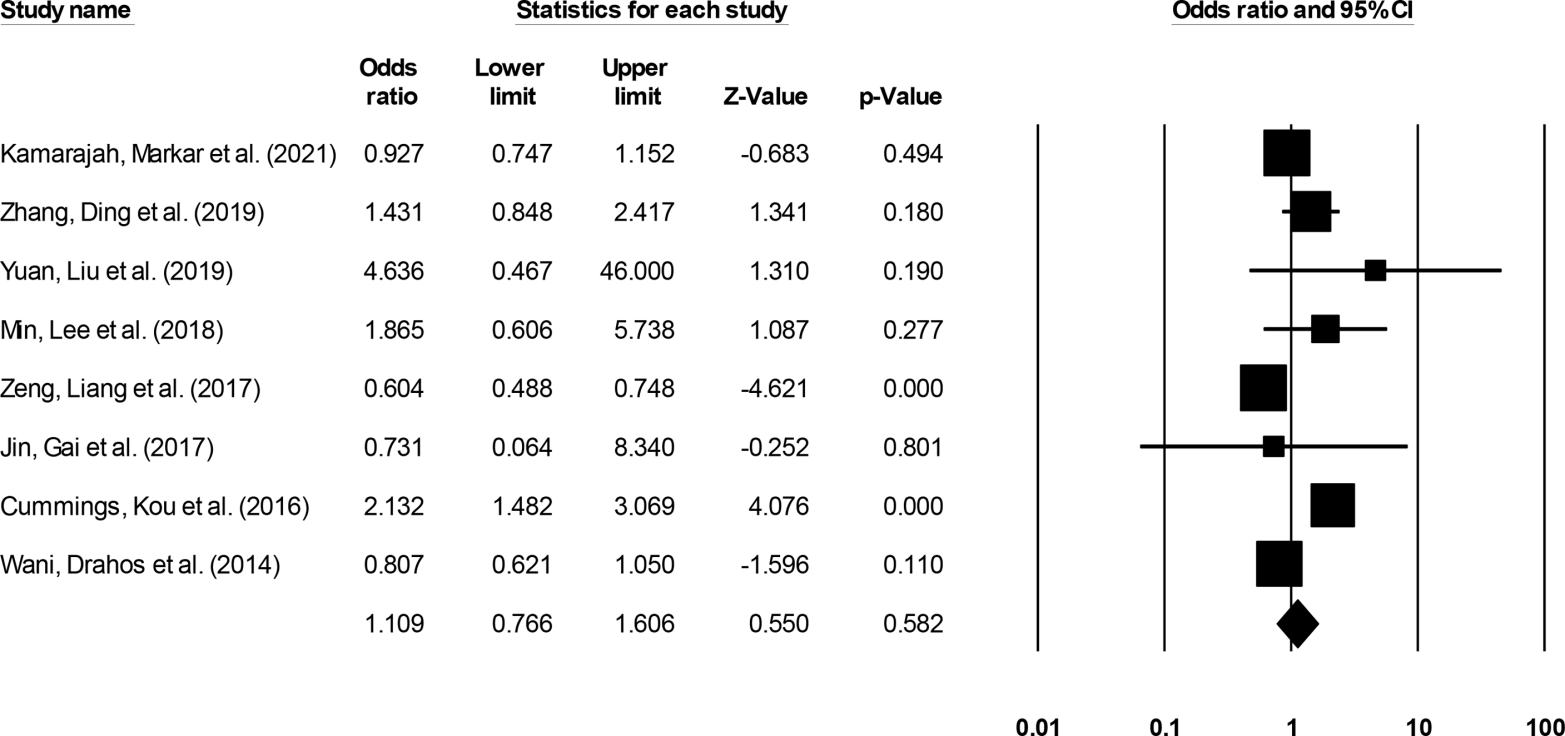
The forest plot for studies evaluating the short-term survival outcome in patients with superficial esophageal squamous cancer undergoing endoscopic submucosal dissection or esophagectomy. The odds ratios are presented as black boxes whereas 95% confidence intervals are presented as whiskers. A higher odds ratio represents higher odds of short-term survival outcome for patients receiving endoscopic submucosal dissection, and a lower odds ratio represents higher odds of short-term survival outcome for patients receiving esophagectomy.

Short-term (≤3 year) incidence was reported by eight studies (14, 15, 18, 22, 29–32). We noted no significant differences in the odds for short-term survival outcome in patients undergoing ESD relative to patients undergoing ESO (**Figure 9**) (1.10, 95% CI: 0.76 to 1.60, p = 0.58) with minimal heterogeneity (I^2^: 14.5%).

#### Long-Term Survival

**Figure 10:**
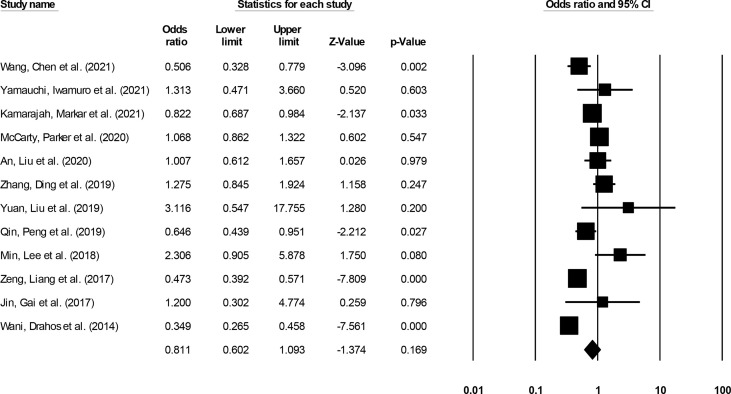
The forest plot for studies evaluating the long-term survival outcome in patients with superficial esophageal squamous cancer undergoing endoscopic submucosal dissection or esophagectomy. The odds ratios are presented as black boxes whereas 95% confidence intervals are presented as whiskers. A higher odds ratio represents higher odds of long-term survival outcome for patients receiving endoscopic submucosal dissection, and a lower odds ratio represents higher odds of long-term survival outcome for patients receiving esophagectomy.

Long-term (>3-year) incidence was reported by 12 studies (14–20, 22, 24, 28, 30, 32). We noted no significant differences in the odds for long-term survival outcome in patients undergoing ESD relative to patients undergoing ESO (**Figure 10**) (0.81, 95% CI: 0.60 to 1.09, p = 0.16) with minimal heterogeneity (I^2^: 12.4%).

## Discussion

This systematic review and meta-analysis provides comprehensive evidence showing the reduced procedure and hospital stay duration alongside reduced risks of complications and all-cause mortality in superficial esophageal squamous cancer patients undergoing ESD as compared to ESO. We also observed that neither the short-/long-term survival rates nor the risks of recurrence were different between either of the therapeutic interventions.

Conventionally, the management of superficial squamous esophageal cancer is carried out by the radical ESO (4). However, owing to the high morbidity- and mortality-related constraints imposed by this procedure, the literature has recommended the use of ESD instead (8, 9). Yamauchi, Iwamuro et al. (20) mentioned that ESD’s application could be more effective in lesions limited to the lamina propria or the epithelial lining of the mucosal membrane as these lesions are seldom associated with lymph-node metastasis.

In our present meta-analysis, we noted that indeed the application of ESD was beneficial relative to ESO in terms of reducing the intervention-related postoperative morbidity, i.e., duration of procedure and hospital stay. Gong, Yue et al. (25) mentioned that because of its less invasive nature, the ESD group also accounted for lesser costs when compared to the patients undergoing ESO (ESD: $3673, ESO: $22272). Similarly, Zhang, Ding et al. (15) also reported a shorter procedure duration in the ESO group and associated this reduction in duration with the lesser events of postoperative adverse events. The authors reported that both fatal (i.e., ESD: 0.3%, ESO: 1.5%) and non-fatal (i.e., ESD: 15.2%, ESO: 27.7%) adverse events were reduced in patients undergoing ESD. Another study included in our review confirmed these findings and reported significantly lower rates of complications in the ESD (18.5%) group relative to the ESO group (55.5%) (29) We also support these findings, as in our meta-analysis we too observed significantly reduced events of all-cause mortality (0.56) and postoperative complications (0.35) in patients undergoing ESD as compared to ESO.

Furthermore, we also attempted to develop a consensus regarding the outcomes of short-term survival, long-term survival, and risks of recurrence between ESD and ESO. We observed that both the risks of recurrence (0.95) and short-term (1.10) and long-term (0.81) survival rates were insignificantly different between either of the intervention groups. This is in line with previously published meta-analysis findings (24). Nonetheless, despite observing similar survival outcomes between patients undergoing ESD and ESO, Yamauchi, Iwamuro et al. (20) recommended the use of ESD for managing esophageal squamous cell carcinoma. The authors attributed this recommendation to the lower levels and severity of postoperative complications observed in their ESD group. We too support this recommendation based on the large number of patients experiencing esophageal squamous cell carcinoma being elderly, and thus, any severe postoperative complication induced after ESO could be life threatening.

In terms of potential clinical implications of our findings, it can be interpreted that clinicians could opt for ESD rather than ESO for managing superficial esophageal squamous cancer. This is also based on the fact that ESD accounts for reduced procedure and hospital stay duration alongside reduced risks of complications and all-cause mortality in superficial esophageal squamous cancer patients. Additionally, although not evaluated in this study, it can also be interpreted from the existing studies that ESD has a higher cost-effectiveness as compared to ESO. Although this study is novel, it faces several challenges. First, the data concerning the distribution of T staging among the included studies and among the ESD/ESO groups were not available for the included studies. Therefore, it cannot be confirmed whether this variable was homogeneously distributed among the two groups. Second, all the studies included in this review were of retrospective nature, thus they might be subject to selection and performance bias. Third, it is well known that ESD especially in the esophagus is a highly sophisticated and technically demanding procedure and since all the included studies were conducted by experts in the field, this might make its application in clinical practice cumbersome. Fourth, the analyses of short-term and long-term survival rates were heterogeneous because different studies had reported the survival outcomes at different follow-up periods. For instance, Wani, Drahos et al. (32) reported the short-term follow-up at 2 years, whereas Min, Lee et al. (29) reported the follow-up at 3 years. Although we did not observe heterogeneity (i.e., I^2^) in either of the analyses, we recommend that the reader interpret the results with caution. Second, we acknowledge that the relative paucity of data within eligible studies may limit our understanding of the comparative differences for the outcome of procedure duration (i.e., four studies) between ESD and ESO. Therefore, a type II error cannot be ruled out. We recommend future cohort, case–control studies to reevaluate the outcomes of procedure duration and survival rate among patients undergoing ESD and ESO, as it would help strengthen the available data.

We herein provide evidence regarding the overall morbidity- and mortality-related risks associated with ESD and ESO. These findings can potentially aid in developing best practice guidelines for selecting an optimal treatment approach for managing superficial esophageal squamous cancer.

## Data Availability Statement

Publicly available datasets were analyzed in this study. These data can be found here: PubMed Central, Cochrane library, EMBASE, and MEDLINE databases from inception until September 2021 for relevant publications.

## Author Contributions

ZL conceived and designed the study. ZL and RZ were involved in literature search and data collection. ZL and RZ analyzed the data. ZL wrote the paper. RZ reviewed and edited the manuscript. All authors contributed to the article and approved the submitted version.

## Conflict of Interest

The authors declare that the research was conducted in the absence of any commercial or financial relationships that could be construed as a potential conflict of interest.

## Publisher’s Note

All claims expressed in this article are solely those of the authors and do not necessarily represent those of their affiliated organizations, or those of the publisher, the editors and the reviewers. Any product that may be evaluated in this article, or claim that may be made by its manufacturer, is not guaranteed or endorsed by the publisher.
