# Supplementary material for: Endoscopic Submucosal Dissection vs. Surgery for Superficial Esophageal Squamous Cancer: A Systematic Review and Meta-Analysis
Source: Front Oncol. 2022 Apr 21;12:816832. doi: 10.3389/fonc.2022.816832 (PMC9068956; doi:10.3389/fonc.2022.816832)
Supplement: Supplementary file 1 [file Table_1.docx]

**Sample search strategy**

| Database | EMBASE |
| --- | --- |
| Strategy | #1 AND #2 AND #3 AND #4 |
| #1 | (‘Squamous cell carcinoma’ OR ‘Carcinoma epidermoid’ OR ‘Carcinoma planocellular’ OR ‘Carcinoma squamous’)/de OR (Squamous cell carcinoma OR Carcinoma epidermoid OR Carcinoma planocellular OR Carcinoma squamous); ti,ab |
| #2 | (‘Esophageal cancer’ OR ‘cancer of esophagus’ OR ‘esophagus cancer’ OR ‘esophageal neoplasm’)/de OR (Esophageal cancer OR cancer of esophagus OR esophagus cancer OR esophageal neoplasm); ti,ab |
| #3 | (‘Esophagectomy’ OR ‘esophageal cancer’ OR ‘endoscopic submucosal dissection’)/de OR (Esophagectomy OR esophageal cancer OR endoscopic submucosal dissection); ti,ab |
| #4 | (‘morbidity’ OR ‘mortality’ OR ‘procedure time’ OR ‘hospital stay’ OR ‘metastasis’ OR ‘complications’ OR ‘recurrence’ OR ‘overall survival’)/de OR (morbidity OR mortality OR procedure time OR hospital stay OR metastasis OR complications OR recurrence OR overall survival); ti,ab |
